# Differences in healthcare expenditure estimates according to statistical approach: A nationwide claims database study on patients with hepatocellular carcinoma

**DOI:** 10.1371/journal.pone.0237316

**Published:** 2020-08-13

**Authors:** Haruhisa Fukuda, Daisuke Sato, Kensuke Moriwaki, Haku Ishida

**Affiliations:** 1 Department of Health Care Administration and Management, Kyushu University Graduate School of Medical Sciences, Fukuoka, Japan; 2 Center for Next Generation of Community Health, Chiba University Hospital, Chiba, Japan; 3 Comprehensive Unit for Health Economic Evidence Review and Decision Support, Ritsumeikan University, Kyoto, Japan; 4 Department of Medical Informatics & Decision Sciences, Yamaguchi University, Yamaguchi, Japan; University of South Carolina College of Pharmacy, UNITED STATES

## Abstract

**Aim:**

Disease-associated healthcare expenditures are generally calculated using matched comparisons or regression-based analyses, but little is known about their differences in estimates. This aim of this study was to compare the differences between disease-associated healthcare expenditures estimated using these 2 methods.

**Methods:**

In this retrospective cohort study, a matched comparison was first conducted by matching cases with controls using sex, age, and comorbidities to estimate disease-associated expenditures. The cases were then used in a fixed-effects analysis that compared expenditures before and after disease occurrence. The subjects were adults (≥20 years) with primary hepatocellular carcinoma (HCC) who underwent treatment (including surgical resection, locoregional therapy, transcatheter arterial chemoembolization, and transarterial embolization) at a Japanese hospital between April 2010 and March 2018. We calculated the total healthcare expenditures per patient per month according to treatment and disease phase (initial, continuing, and terminal).

**Results:**

There were 14,923 cases in the initial/continuing phases and 15,968 cases in the terminal phase. In the initial/continuing phases, 3,552 patients underwent surgical resection only, with HCC-associated expenditures of $5,555 according to the matched comparison and $5,889 according to the fixed-effects analysis (proportional difference: 94.3%). The initial phase expenditures were approximately 9% higher in the fixed-effects analysis, whereas the continuing phase expenditures were approximately 7% higher in the matched comparison. The expenditures in the terminal phase were 93.1% higher in the fixed-effects analysis.

**Conclusions:**

The 2 methods produced similar estimates of HCC-associated healthcare expenditures in the initial/continuing phases. However, terminal phase expenditures were substantially different between the methods.

## Introduction

Hepatocellular carcinoma (HCC) is the sixth-most prevalent cancer and the second-most common cause of cancer death worldwide. The incidence of HCC continues to increase in numerous countries [1–4], and HCC-related mortality is rising in the US [5]. Similar epidemiological trends have also been observed in Japan [6].

Increases in HCC incidence manifest as corresponding increases in healthcare expenditures. In order to anticipate the future economic cost of this disease relative to non-HCC patients from the public insurer’s perspective, policymakers must have access to accurate estimates of the expenditures incurred during HCC treatment. For example, cost-effectiveness analyses of new technologies to reduce HCC incidence (e.g., direct-acting antiviral therapy for hepatitis C virus [HCV] infections) [7–11] and surveillance programs to allow early detection of HCC [12–17] are important parameters of such expenditures.

Over the past decade, studies from various countries have produced estimates of HCC-associated healthcare expenditures [4,18–24]. However, the majority of these estimates are based on observational studies, and may therefore be influenced by the statistical estimation method used. Many such estimates are derived from matched comparisons of HCC and non-HCC patients [4,18–20,23–28], and few studies have employed regression-based approaches to compare the expenditures in HCC patients before and after disease occurrence [24,29,30]. Matched comparisons are unable to account for variations in patient characteristics that are not specified in the matching criteria. In contrast, analytical approaches in which individual patients act as their own controls can adjust for inherent patient characteristics, but are unable to account for time-dependent changes in the severity of comorbidities after disease occurrence. Both approaches therefore have inherent benefits and limitations. However, the magnitude of differences in the estimates of HCC-associated healthcare expenditures between these approaches has yet to be examined.

In this study, we used an insurance claims database that encompassed almost all HCC cases in Japan to estimate the HCC-associated healthcare expenditures through (1) a matched comparison of HCC and non-HCC patients and (2) a fixed-effects analysis of HCC patients. The expenditures were estimated and compared according to treatment type and disease phase.

## Methods

### Data

This study was conducted using the National Database of Health Insurance Claims and Specific Health Checkups of Japan (NDB), which compiles and stores nationwide insurance claims data for all medical goods and services provided under the national health insurance system. Anonymized claims data from April 2010 to March 2018 were extracted for analysis. Previous studies on HCC-associated expenditures have generally used population-based data, such as Surveillance, Epidemiology, and End Results (SEER)-Medicare linked data, Nationwide Inpatient Sample data, and Ontario Cancer Registry-linked administrative data [4,18–20,22]. In contrast, the NDB provides a near-comprehensive database that covers almost all residents in Japan. The NDB includes information on basic patient characteristics, medical services, prescribed drugs, medical devices, recorded diagnoses, and expenditures. However, it lacks clinical information such as laboratory results and cancer staging. As the NDB continues to collect data on insurance-covered healthcare provided to patients at all medical facilities in Japan, the use of healthcare in patients can be tracked over time and across providers.

This study was approved by the Kyushu University Institutional Review Board for Clinical Research (Approval Number 30–149). Because all patient records were de-identified prior to analysis, the review board waived the requirement for informed consent. All data were fully anonymized by the NDB before receipt by the authors. Data from April 2010 to March 2018 were accessed.

### Case patients

The subjects were adult patients (aged ≥20 years) who developed primary HCC during the study period. We identified patients with no recorded diagnosis of any cancer (including HCC) for at least one year before the index HCC diagnosis (i.e., the first recorded diagnosis of HCC between April 2010 and March 2018). This allowed us to exclude cases in which HCC was due to metastasis from other sites, and focus on primary HCC patients.

### Healthcare expenditure measurements

The outcome measure was HCC-associated healthcare expenditure per patient per month (PPPM) from the perspective of the public insurer (i.e., the payer). The NDB provides monthly claims data from each healthcare provider. Healthcare expenditures include HCC-associated expenditures (i.e., fees for consultations, hospitalizations, laboratory tests, prescriptions, medical device use, and surgeries for HCC diagnosis and treatment) and unrelated expenditures, and were estimated according to treatment type and disease phase. The treatments for HCC were: (1) surgical resection; (2) locoregional therapy (LRT), including percutaneous ethanol injection, percutaneous microwave coagulation therapy, and radiofrequency ablation; (3) transcatheter arterial chemoembolization (TACE); (4) transarterial embolization (TAE); (5) molecular targeted therapy (sorafenib); (6) systemic chemotherapy; and (7) radiotherapy. We also examined common combinations of these treatments.

**Fig 1:**
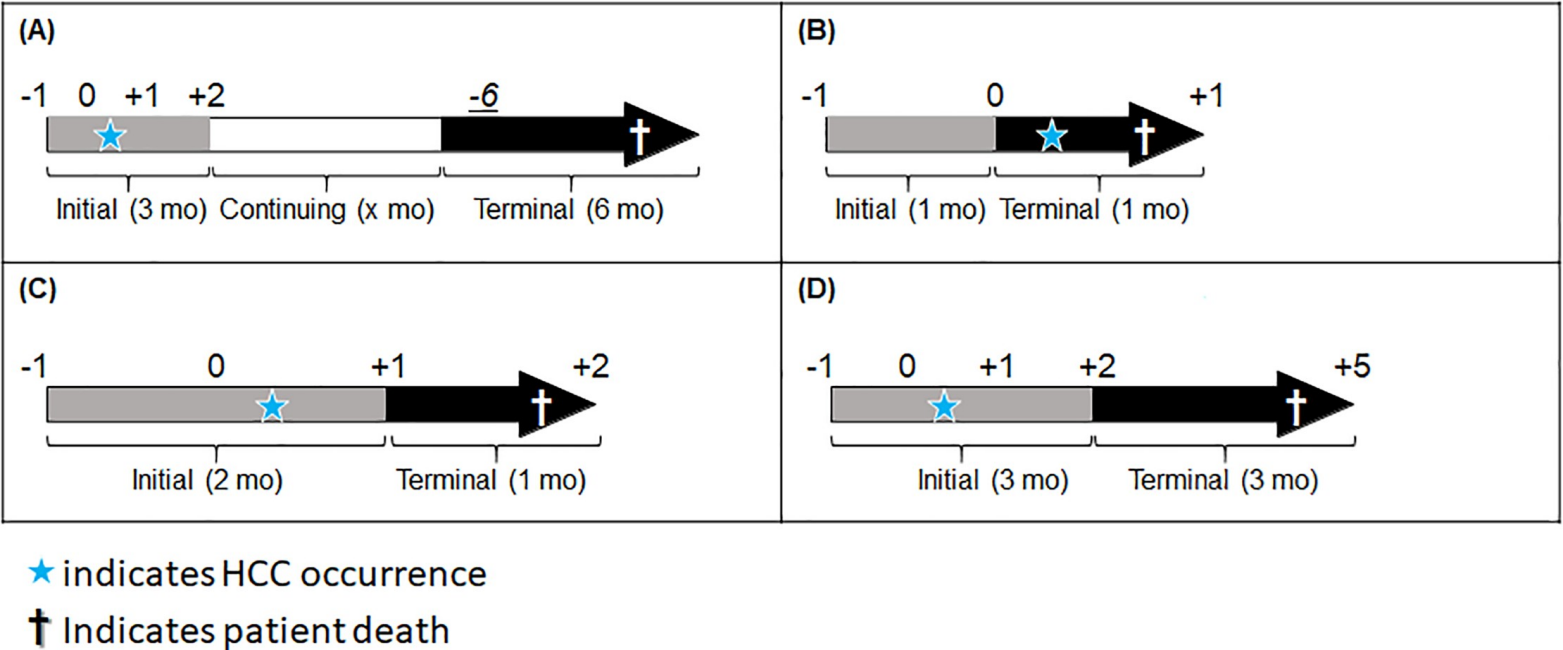
Definitions of the initial, continuing, and terminal phases. (A) Standard pattern, (B) Exception 1: Death during the index month, (C) Exception 2: Death 1 month after the index month, (D) Exception 3: Death 5 months after the index month.

Disease phases were categorized into the initial phase, continuing phase, and terminal phase [19]. **Fig 1** shows the classification criteria for these phases. The initial phase was a 3-month period consisting of the index month (when primary HCC was first diagnosed), the preceding month, and the following month. The continuing phase comprised the period beginning 2 months after the index month until 7 months before death. The terminal phase comprised the 6 months before death. However, if a patient died during the index month, the initial phase included only the month preceding the index month, and the terminal phase included only the index month; there was no continuing phase for these patients. If death occurred within 7 months of the index month, the initial phase remained the same, but the terminal phase was set as 2 months after the index month until 6 months (or less) before death; there was no continuing phase for these patients.

Recurrent HCC was identified as a new diagnosis of HCC after a patient experienced 7 months or more without any anticancer therapy after the initial treatment. As these recurrent cases would have undue influence on expenditure estimates, they were excluded from analysis.

### Statistical analysis

**Fig 2:**
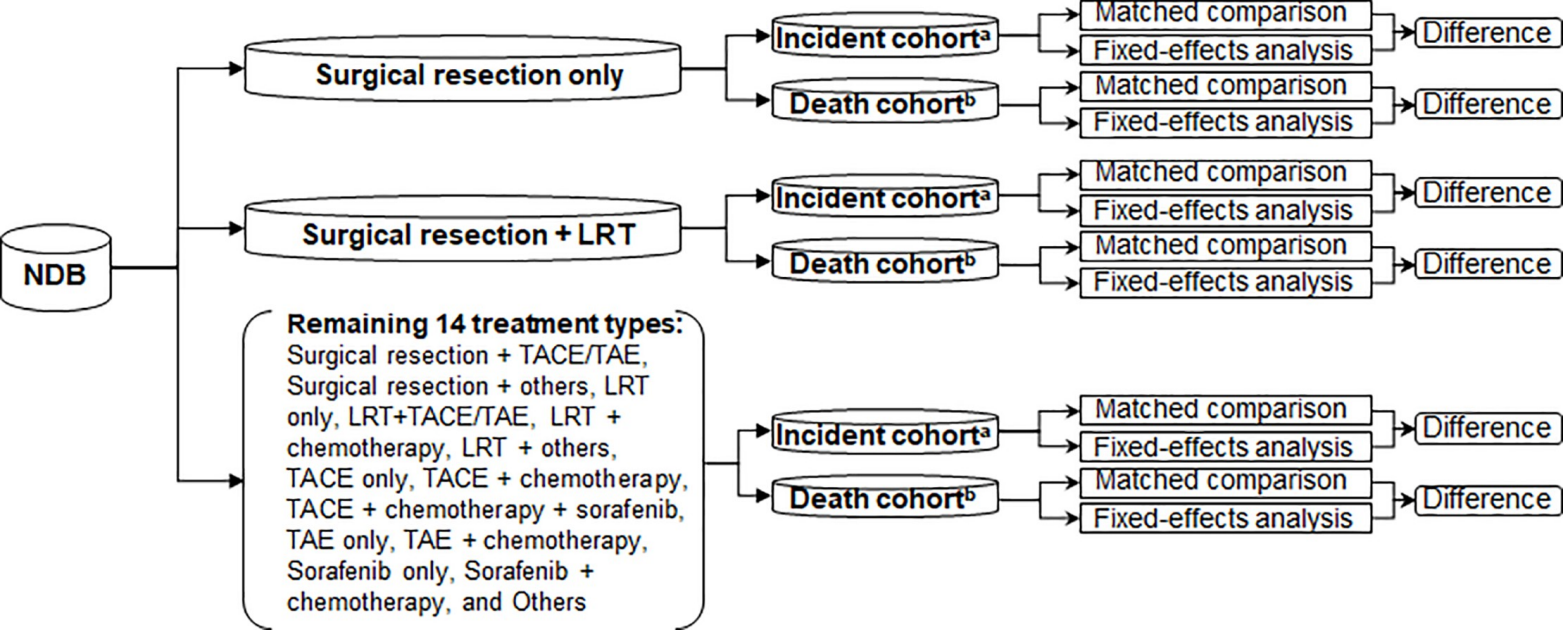
Study design. ^**a**^Using estimates from the initial and continuing phases. ^**b**^Using estimates from the terminal phase. Abbreviations: LRT, locoregional therapy; NDB, National Database of Health Insurance Claims and Specific Health Checkups of Japan; TACE, transcatheter arterial chemoembolization; TAE, transarterial embolization.

Healthcare expenditures include both HCC-associated expenditures and unrelated expenditures. Accordingly, the unrelated expenditures must be excluded to quantify the expenditures associated with HCC treatment. To do so, we employed 2 statistical approaches: a matched comparison and a fixed-effects analysis. The HCC cases used in the matched comparison were also used as subjects in the fixed-effects analysis. The overall study design is illustrated in **Fig 2**. In order to compare the differences in estimates from the matched comparison and fixed-effects models, we calculated the proportional differences and absolute differences between the 2 approaches using the following formulae:

$$Proportional\;difference=1-\frac{Matched\;comparison\;estimates}{Fixed\;effects\;analysis\;estimates}$$

Absolutedifference=Fixedeffectsanalysisestimates−Matchedcomparisonestimates

In order to account for the influence of periodic revisions to the reimbursement system, the revision rates from each year of study (relative to 2018) were applied (1.019 for 2010–2011, 1.004 for 2012–2013, 1.1001 for 2014–2015, and 0.9916 for 2016–2017). The 2017 purchasing power parity rate ($1.00 = 102.5 yen) was used to convert Japanese yen to US dollars. All statistical analyses were performed using Stata 15.1 (Stata Corp., College Station, Texas, USA).

#### Matched comparison

For this method, HCC patients were matched based on a specified set of patient characteristics with non-HCC patients randomly extracted from the NDB. Similar to the approach used in Thein et al. [19], we assigned separate cohorts to estimate expenditures in the initial and continuing phases (designated the incident cohort) and the terminal phase (designated the death cohort).

The matching variables for the incident cohort were age (5-year intervals), sex (male or female), and 15 conditions (yes or no) from the Charlson comorbidity index (CCI) excluding tumors and metastatic solid tumors. For each case patient, we identified all candidate control patients who received outpatient or inpatient care at a medical facility during the same index month, had identical matching variables, and had no history of HCC. One control was randomly selected from among these candidates for each case, and each identified control was removed from the pool of candidate controls for other cases. If a control could not be found using this method, the matching period was expanded to include patients who received care during the month closest to the index month (up to a maximum of one year) of the case. For the death cohort, we matched each case with a randomly selected control who had died during the same month, had identical matching variables, and had no history of HCC.

We included only HCC cases for whom an eligible non-HCC control could be identified using these criteria, and conducted a paired comparison analysis of expenditures. The differences in healthcare expenditure PPPM between the case and control groups were calculated.

#### Fixed-effects analysis

The second method involved the use of fixed-effects models where individual patients acted as their own controls. In each patient, the month in which HCC treatment began was designated the case month, and the preceding month was designated the control month. The dependent variable was healthcare expenditure PPPM. Time-dependent factors (age and CCI) were included as covariates, and time-invariant patient characteristics (including sex) were included as fixed effects. In the matched comparison described above, we used different patients for the initial and continuing phases (incident cohort) and the terminal phase (death cohort). To enable direct comparisons between the estimation methods, we also developed separate fixed-effects models for the incident and death cohorts. In this study, each patient’s study period was divided into the initial phase (beginning from the index month), continuing phase, and terminal phase (ending in the month of death). During these phases, months in which a patient had no healthcare utilization were still included in analysis as HCC treatment months despite not having any HCC-associated expenditures.

## Results

We identified 25,871 patients in the incident cohort and 23,967 patients in the death cohort who had new diagnoses of primary HCC, had undergone any of the specified treatments, and had no recurrent HCC between April 2010 and March 2018. Among these, 14,923 cases in the incident cohort and 15,968 cases in the death cohort could be matched with a corresponding control (**Table 1**). When compared with the unmatched cases, the matched cases in the incident cohort were older (71.8 years vs. 68.1 years, *P* < 0.001), had a higher proportion of women (34.9% vs. 28.4%, *P* < 0.001), and had a higher CCI score (6.4 vs. 4.8, *P* < 0.001). The matched cases in the death cohort were older (74.2 years vs. 72.2 years, *P* < 0.001), but had a lower proportion of women (30.1% vs. 33.2%, *P* < 0.001) and a lower CCI score (6.8 vs. 8.8, *P* < 0.001) than the unmatched cases. As the controls were selected based on having the same patient characteristics as the cases, the characteristics of the control group were identical to those of the matched cases group. **Table 2** summarizes the distribution of patient numbers (according to disease phase) and characteristics among the treatment types. The most common type of treatment was surgical resection only (23.8% of all cases), followed by TACE only (15.2%) and LRT only (13.7%).

**Table 1:** Characteristics of matched and unmatched hepatocellular carcinoma patients in the incident and death cohorts.

	Incident Cohort			Death Cohort		
	Unmatched Cases (n = 10,948)	Matched Cases (n = 14,923)	*P*	Unmatched Cases (n = 7,999)	Matched Cases (n = 15,968)	*P*
Age, mean [SD]	68.1 [10.9]	71.8 [10.6]	<0.001	72.2 [11.1]	74.2 [9.6]	<0.001
Female	3102 (28.4%)	5201 (34.9%)	0.000	2653 (33.2%)	4804 (30.1%)	<0.001
CCI score, mean [SD]	4.8 [1.8]	6.4 [2.4]	<0.001	8.8 [2.1]	6.8 [2.3]	<0.001
Myocardial infarction, n (%)	182 (1.7%)	1050 (7.0%)	<0.001	723 (9.0%)	318 (2.0%)	<0.001
Congestive heart failure, n (%)	1514 (13.8%)	4690 (31.4%)	<0.001	3176 (39.7%)	4419 (27.7%)	<0.001
Peripheral vascular disease, n (%)	919 (8.4%)	2984 (20.0%)	<0.001	1672 (20.9%)	962 (6.0%)	<0.001
Cerebrovascular disease, n (%)	1465 (13.4%)	3987 (26.7%)	<0.001	2514 (31.4%)	2857 (17.9%)	<0.001
Dementia, n (%)	149 (1.4%)	1204 (8.1%)	<0.001	1009 (12.6%)	1201 (7.5%)	<0.001
Chronic obstructive pulmonary disease, n (%)	1846 (16.9%)	3835 (25.7%)	<0.001	2658 (33.2%)	3571 (22.4%)	<0.001
Rheumatic disease, n (%)	206 (1.9%)	836 (5.6%)	<0.001	545 (6.8%)	149 (0.9%)	<0.001
Peptic ulcer, n (%)	4759 (43.5%)	7074 (47.4%)	<0.001	4553 (56.9%)	8134 (50.9%)	<0.001
Mild liver disease, n (%)	9752 (89.1%)	13111 (87.9%)	0.003	7585 (94.8%)	14434 (90.4%)	<0.001
Diabetes, n (%)	2366 (21.6%)	5555 (37.2%)	<0.001	3056 (38.2%)	2238 (14.0%)	<0.001
Diabetes with complications, n (%)	1197 (10.9%)	3449 (23.1%)	<0.001	2193 (27.4%)	803 (5.0%)	<0.001
Paraplegia and hemiplegia, n (%)	61 (0.6%)	351 (2.4%)	<0.001	351 (4.4%)	85 (0.5%)	<0.001
Renal disease, n (%)	301 (2.8%)	1917 (12.9%)	<0.001	1938 (24.2%)	1329 (8.3%)	<0.001
Moderate/severe liver disease, n (%)	1334 (12.2%)	4569 (30.6%)	<0.001	6359 (79.5%)	8346 (52.3%)	<0.001
Acquired immune deficiency syndrome, n (%)	13 (0.1%)	21 (0.1%)	0.630	15 (0.2%)	n.r.	<0.001

CCI, Charlson comorbidity index; n.r., Not reportable in accordance with the data dissemination standards of the NDB (Cells with <9 cases cannot be reported).

**Table 2:** Distribution of hepatocellular carcinoma patient numbers and characteristics among the treatment types.

	Disease Phase			Female	Age^†^	CCI^†^
Treatment Type	Initial	Continuing	Terminal			
Surgical resection only	3552 (23.8%)	3328 (24.4%)	724 (4.5%)	1,050 (29.6%)	68.9 [9.8]	5.6 [2.1]
Surgical resection + LRT	49 (0.3%)	47 (0.3%)	12 (0.1%)	12 (24.5%)	70.5 [10.7]	6.1 [2.5]
Surgical resection + TACE/TAE	208 (1.4%)	202 (1.5%)	88 (0.6%)	51 (24.5%)	70.4 [9.6]	5.8 [2.1]
Surgical resection + others	511 (3.4%)	487 (3.6%)	478 (3.0%)	147 (28.8%)	68.8 [9.5]	6.0 [2.2]
LRT only	2044 (13.7%)	1895 (13.9%)	652 (4.1%)	891 (43.6%)	71.7 [10.7]	6.6 [2.4]
LRT+TACE/TAE	720 (4.8%)	688 (5.0%)	250 (1.6%)	278 (38.6%)	71.2 [10.0]	6.5 [2.3]
LRT + chemotherapy	240 (1.6%)	226 (1.7%)	221 (1.4%)	112 (46.7%)	71.4 [9.8]	6.9 [2.5]
LRT + others	142 (1.0%)	132 (1.0%)	215 (1.3%)	47 (33.1%)	71.8 [10.5]	6.6 [2.5]
TACE only	2264 (15.2%)	1932 (14.2%)	1960 (12.3%)	796 (35.2%)	74.9 [10.2]	6.8 [2.3]
TACE + chemotherapy	248 (1.7%)	235 (1.7%)	421 (2.6%)	86 (34.7%)	72.2 [10.7]	6.6 [2.4]
TACE + chemotherapy + sorafenib	90 (0.6%)	86 (0.6%)	169 (1.1%)	19 (21.1%)	68.3 [10.2]	6.0 [2.5]
TAE only	78 (0.5%)	61 (0.4%)	290 (1.8%)	25 (32.1%)	80.5 [11.8]	6.3 [2.6]
TAE + chemotherapy	36 (0.2%)	32 (0.2%)	137 (0.9%)	n.r.	72.7 [10.9]	6.2 [2.3]
Sorafenib only	191 (1.3%)	157 (1.2%)	797 (5.0%)	49 (25.7%)	71.7 [10.4]	6.3 [2.4]
Sorafenib + chemotherapy	274 (1.8%)	237 (1.7%)	812 (5.1%)	56 (20.4%)	69.5 [10.5]	6.0 [2.2]
Others	4276 (28.7%)	3893 (28.5%)	8742 (54.7%)	1,574 (36.8%)	73.1 [10.7]	6.8 [2.4]
Total	14,923	13,638	15,968	5,201 (34.9%)	71.8 [10.6]	6.4 [2.4]

^†^ Values were obtained from the initial phase.

SD, standard deviation; CCI, Charlson comorbidity index; LRT, locoregional therapy; TACE, transcatheter arterial chemoembolization; TAE, transarterial embolization; n.r., Not reportable in accordance with the data dissemination standards of the NDB (Cells with <9 cases cannot be reported).

**Table 3** shows the results of the matched comparison of HCC cases and non-HCC controls. We estimated the mean net healthcare expenditure PPPM of the cases by subtracting the difference with the expenditure of the controls according to treatment type and disease phase. Surgical resection was a component of the most expensive treatment type (surgical resection + others) during the initial phase, and the healthcare expenditures associated with surgical resection only were estimated to be $5,555 during this phase. In contrast, TACE was a component of the most expensive treatment type (TACE + chemotherapy + sorafenib) during the continuing phase, and the healthcare expenditures associated with TACE only were estimated to be $498 during this phase. In the terminal phase, treatments that included surgical resection tended to be more expensive than the other treatments; the healthcare expenditures associated with surgical resection only were estimated to be $1,484 during this phase.

**Table 3:** Mean net healthcare expenditures associated with hepatocellular carcinoma estimated using the matched comparison.

Treatment Type	Matched Cases			Matched Controls			Net Expenditure		
	Initial	Continuing	Terminal	Initial	Continuing	Terminal	Initial	Continuing	Terminal
Surgical resection only	7136 (6996–7277)	971 (956–986)	5918 (5643–6193)	1581 (1516–1646)	888 (873–903)	4434 (4258–4610)	5555 (5404–5706)	83 (62–104)	1484 (1163–1805)
Surgical resection + LRT	6448 (5328–7568)	1429 (1241–1616)	1951 (1388–2514)	1708 (1140–2276)	1098 (965–1231)	2775 (1949–3601)	4740 (3511–5969)	330 (117–544)	3570 (1805–5335)
Surgical resection + TACE/TAE	6881 (6330–7432)	1626 (1517–1734)	5510 (4839–6182)	1630 (1366–1894)	840 (770–911)	4063 (3604–4523)	5251 (4646–5855)	785 (657–913)	1447 (639–2254)
Surgical resection + others	7842 (7429–8255)	1460 (1410–1510)	5974 (5671–6278)	1660 (1475–1846)	831 (797–866)	4169 (3962–4375)	6182 (5740–6623)	629 (570–688)	1806 (1441–2171)
LRT only	2999 (2920–3078)	1349 (1317–1380)	3514 (3387–3641)	1669 (1582–1755)	941 (918–965)	3962 (3811–4113)	1330 (1216–1445)	407 (368–446)	-448 (-636—260)
LRT+TACE/TAE	4338 (4164–4511)	1542 (1484–1600)	3531 (3333–3729)	1619 (1472–1766)	948 (907–988)	3778 (3542–4014)	2719 (2496–2942)	594 (525–664)	-247 (-541-47)
LRT + chemotherapy	4235 (3935–4535)	1048 (996–1100)	3368 (3179–3558)	1570 (1335–1806)	862 (789–936)	3872 (3610–4135)	2665 (2288–3041)	186 (99–273)	-504 (-818—189)
LRT + others	4176 (3756–4595)	2532 (2407–2658)	4604 (4356–4851)	1520 (1236–1803)	973 (888–1058)	4272 (4009–4534)	2656 (2171–3141)	1559 (1409–1710)	332 (-22-685)
TACE only	4222 (4122–4321)	1550 (1515–1585)	3935 (3857–4012)	1815 (1729–1902)	1052 (1023–1081)	4065 (3971–4159)	2406 (2278–2534)	498 (453–543)	-130 (-248—13)
TACE + chemotherapy	4333 (4040–4625)	2106 (2011–2202)	3964 (3816–4111)	2235 (1933–2538)	1130 (1056–1204)	3993 (3820–4167)	2098 (1689–2506)	976 (857–1096)	-30 (-253-194)
TACE + chemotherapy + sorafenib	4144 (3667–4622)	3219 (3062–3376)	4357 (4141–4574)	1537 (1198–1877)	767 (673–861)	4313 (3958–4668)	2607 (2050–3164)	2452 (2276–2628)	45 (-373-462)
TAE only	5232 (4504–5961)	778 (681–876)	5782 (5407–6157)	2177 (1641–2714)	931 (809–1052)	4035 (3735–4336)	3055 (2216–3894)	-152 (-306-1)	1747 (1277–2216)
TAE + chemotherapy	4899 (3781–6017)	1050 (892–1209)	4311 (3966–4656)	2108 (1511–2705)	968 (750–1185)	3613 (3296–3931)	2791 (1589–3994)	83 (-182-347)	698 (236–1159)
Sorafenib only	4282 (4003–4560)	1312 (1230–1394)	4290 (4174–4406)	1324 (1000–1648)	572 (521–624)	4208 (4041–4375)	2958 (2537–3378)	740 (644–835)	82 (-118-282)
Sorafenib + chemotherapy	4373 (4096–4649)	1648 (1575–1721)	4348 (4241–4456)	1742 (1509–1975)	698 (647–749)	3938 (3796–4080)	2631 (2269–2993)	950 (864–1037)	410 (233–588)
Others	4638 (4545–4731)	1011 (999–1023)	4072 (4031–4113)	1775 (1713–1836)	802 (790–813)	3948 (3903–3993)	2863 (2755–2972)	209 (193–225)	124 (65–183)
Total	5057 (5003–5110)	1175 (1166–1184)	4214 (4182–4247)	1703 (1671–1736)	870 (863–878)	4006 (3973–4040)	3353 (3292–3414)	305 (294–316)	208 (163–253)

Values are presented as mean USD [95% confidence intervals].

LRT, locoregional therapy; TACE, transcatheter arterial chemoembolization; TAE, transarterial embolization.

Using the HCC case patients from the matched comparison, we compared the mean net healthcare expenditure PPPM before and after HCC onset estimated by the fixed-effects models (**Table 4**). The most expensive treatments in the initial and terminal phases included surgical resection. The healthcare expenditures associated with surgical resection only were estimated to be $5,889 and $4,744 in the initial phase and terminal phase, respectively. In contrast, the most expensive treatment in the continuing phase included TACE, and the healthcare expenditures associated with TACE + chemotherapy were estimated to be $1,113 during this phase.

**Table 4:** Mean net healthcare expenditures associated with hepatocellular carcinoma estimated using the fixed-effects analysis.

	Incidence Cohort			Death Cohort	
	Reference Expenditure	Net Expenditure		Reference Expenditure	Net Expenditure
	Pre-HCC	Initial	Continue	Pre-HCC	Terminal
Surgical resection only	649 (640–658)	5,889 (5,846–5,932)	17 (-5-40)	435 (416–455)	4,744 (4,651–4,836)
Surgical resection + LRT	607 (543–671)	5,244 (4,908–5,581)	545 (357–733)	555 (422–688)	4,786 (4,176–5,397)
Surgical resection + TACE/TAE	699 (660–738)	5,343 (5,138–5,549)	413 (302–525)	372 (315–430)	4,361 (4,079–4,643)
Surgical resection + others	635 (607–663)	6,436 (6,308–6,565)	546 (479–613)	367 (341–392)	4,761 (4,637–4,885)
LRT only	775 (764–785)	1,601 (1,547–1,655)	237 (208–266)	577 (562–592)	2,311 (2,246–2,377)
LRT+TACE/TAE	741 (724–757)	2,881 (2,791–2,972)	310 (258–361)	571 (546–596)	2,244 (2,132–2,357)
LRT + chemotherapy	601 (571–631)	2,982 (2,847–3,116)	308 (239–378)	435 (411–458)	2,076 (1,975–2,177)
LRT + others	705 (664–747)	2,763 (2,551–2,974)	1,357 (1,237–1,477)	489 (459–519)	3,317 (3,188–3,447)
TACE only	747 (738–755)	2,658 (2,608–2,709)	274 (244–304)	458 (450–465)	2,689 (2,652–2,725)
TACE + chemotherapy	615 (587–644)	2,897 (2,747–3,047)	1,113 (1,027–1,198)	390 (372–408)	2,898 (2,816–2,980)
TACE + chemotherapy + sorafenib	602 (547–657)	2,854 (2,588–3,119)	2,040 (1,884–2,195)	365 (334–397)	3,245 (3,107–3,382)
TAE only	596 (555–637)	4,016 (3,792–4,240)	-75 (-204-54)	352 (336–369)	4,476 (4,370–4,583)
TAE + chemotherapy	377 (308–446)	3,769 (3,412–4,125)	685 (495–875)	322 (296–348)	3,051 (2,915–3,187)
Sorafenib only	547 (518–576)	2,617 (2,460–2,775)	775 (687–864)	241 (232–250)	3,113 (3,063–3,162)
Sorafenib + chemotherapy	379 (355–403)	2,860 (2,740–2,979)	1,036 (967–1,104)	271 (259–282)	3,125 (3,067–3,184)
Others	542 (535–550)	3,270 (3,235–3,305)	338 (320–355)	311 (307–314)	2,870 (2,852–2,888)
Total	646 (642–650)	3,684 (3,664–3,704)	285 (274–295)	351 (348–354)	3,026 (3,012–3,040)

Values are presented as mean USD [95% confidence intervals].

LRT, locoregional therapy; TACE, transcatheter arterial chemoembolization; TAE, transarterial embolization.

**Table 5** shows the comparison of estimates between the matched comparison and fixed-effects analysis. The results are presented as the proportional and absolute differences between the mean net healthcare expenditure estimates of the matched comparison and the estimates of the fixed-effects analysis. Between the 2 approaches, there were relatively small proportional differences in total expenditures for the initial (91.0%) and continuing phases (107.0%). However, the differences in treatment-level expenditures varied widely for the continuing phase. In general, the initial phase estimates tended to be higher in the fixed-effects analysis, whereas the continuing phase estimates tended to be higher in the matched comparison. The estimates for surgical resection only, TACE only, and TAE only from the matched comparison were substantially (81.8–379.1%) higher than the corresponding estimates from the fixed-effects analysis. When examining the absolute differences in expenditures, the estimates from the matched comparison for surgical resection only, TACE only, and TAE only were $66, $224, and $77 higher than the fixed-effects analysis. However, the estimates in the terminal phase varied widely between the analytical approaches, and the fixed-effects analysis produced a total estimate that was 93.1% (proportional difference) and $2,818 (absolute difference) higher than the matched comparison. The estimates were generally higher in the fixed-effects analysis across the treatment types.

**Table 5:** Differences in healthcare expenditure estimates between the matched comparison and fixed-effects analysis.

	Proportional difference			Absolute Difference		
Treatment	Initial	Continuing	Terminal	Initial	Continuing	Terminal
Surgical resection only	5.7%	-379.1%	68.7%	334	-66	3,260
Surgical resection + LRT	9.6%	39.4%	25.4%	504	215	1,216
Surgical resection + TACE/TAE	1.7%	-90.0%	66.8%	93	-372	2,915
Surgical resection + others	4.0%	-15.1%	62.1%	255	-83	2,955
LRT only	16.9%	-72.0%	119.4%	271	-171	2,759
LRT+TACE/TAE	5.6%	-91.9%	111.0%	162	-285	2,491
LRT + chemotherapy	10.6%	39.8%	124.3%	317	123	2,580
LRT + others	3.9%	-14.9%	90.0%	106	-202	2,986
TACE only	9.5%	-81.8%	104.8%	252	-224	2,819
TACE + chemotherapy	27.6%	12.3%	101.0%	800	136	2,928
TACE + chemotherapy + sorafenib	8.7%	-20.2%	98.6%	247	-412	3,200
TAE only	23.9%	-103.0%	61.0%	961	77	2,730
TAE + chemotherapy	25.9%	87.9%	77.1%	977	603	2,353
Sorafenib only	-13.0%	4.6%	97.4%	-340	36	3,030
Sorafenib + chemotherapy	8.0%	8.2%	86.9%	229	85	2,715
Others	12.4%	38.0%	95.7%	406	128	2,746
Total	9.0%	-7.0%	93.1%	330	-20	2,818

Values are presented as the estimates of the matched comparison relative to the estimates of the fixed-effects analysis. LRT, locoregional therapy; TACE, transcatheter arterial chemoembolization; TAE, transarterial embolization.

## Discussion

In an analysis of almost all primary HCC cases in Japan over 9 years, we compared the estimates of HCC-associated healthcare expenditures using 2 different statistical approaches. The key findings are as follows: when comparing the estimates produced by the commonly used matched comparison approach and the regression-based fixed-effects analysis, we found no considerable differences in the initial and continuing phase estimates. However, there were substantial differences in the terminal phase estimates between the methods. To the best of our knowledge, this is the first study that uses the same HCC patients to quantify the differences in estimates between these analytical approaches. Our study also found that healthcare expenditures varied widely among the different treatments.

These findings can contribute to our understanding of the types of databases that should be used in the estimation of healthcare expenditures. The matched comparison and fixed-effects analysis produced similar estimates in the initial and continuing phases across all treatment types. Because matched comparisons require a sample of control patients and numerous variables for matching, this approach benefits from a relatively large database. In contrast, the use of longitudinal data in the fixed-effects analysis eliminates the need for separate controls and matching variables. However, there were large differences in terminal phase estimates between the 2 methods. The fixed-effects approach compares expenditures in the same patients before and after HCC onset, and therefore cannot accurately provide insight into the expenses incurred during the terminal phase under conditions where HCC had not occurred. Because the majority of patients experience an increase in healthcare expenditures during end-of-life care, the matched comparison approach is more appropriate for this phase.

Although many previous studies have used matched comparisons to estimate HCC-associated healthcare expenditures [4,19,20,23,25–28], there has been a recent increase in studies that use regression models to compare HCC and non-HCC patients [24,29,30]. In an analysis of SEER-Medicare linked data using a 2-part model, Rein et al. estimated that HCC patients had additional expenditures of $2,917 PPPM compared to HCV patients [29]. Lang et al. estimated HCC-associated expenditures to be $2,446 PPPM using the same data source [4]. Menzin et al. estimated that HCC patients had additional expenditures of $2,297 PPPM compared to HCV patients in a matched comparison using a Florida-based Medicaid claims database [27]. In contrast, McAdam-Marx et al. and White et al. reported that HCC is associated with minimum additional expenditures of $3,639 and $7,265, respectively, when compared with non-HCC patients [26,28]; these estimates were markedly higher than those reported in Rein et al. and Menzin et al. [27,29]. The differences may be due to the limitation of the control groups in Rein et al. and Menzin et al. to HCV patients, who would have more severe disease conditions and higher expenditures than general non-HCC patients.

Our analysis estimated HCC-associated healthcare expenditures according to treatment type and disease phase, which was similar to the approach by Thein et al. [19]. In their Canada-based study, they conducted analyses on 2,320 patients in the initial phase, 1,478 patients in the continuing phase, and 1,103 patients in the terminal phase. They reported that HCC was associated with additional healthcare expenditure PPPM of $3,204 in the initial phase, $2,055 in the continuing phase, and $7,776 in the terminal phase [19]. In contrast, our study was conducted using a substantially larger sample of 14,923 patients in the initial phase, 13,638 patients in the continuing phase, and 15,968 patients in the terminal phase. Using the matched comparison approach, we estimated that HCC was associated with additional healthcare expenditure PPPM of $3,353 in the initial phase, $305 in the continuing phase, and $208 in the terminal phase. With the exception of the initial phase, our estimates were markedly different with those of Thein et al. [19]. A possible reason is that liver transplantations are conducted with very low frequency in Japan, and our estimates did not include these costly procedures. The disparities may also be attributable to inherent differences between the healthcare systems of Japan and Canada.

During the continuing phase, HCC patients who had undergone TAE only had a lower mean expenditure than non-HCC patients (as evidenced by the negative net expenditure) in the fixed-effects analysis. There were only 61 eligible patients in the TAE only group during this phase, many of whom did not incur high expenditures. This small sample size may have influenced the relatively low expenditures. Similarly, the matched comparison approach showed negative net expenditures for several treatments in the terminal phase. During that phase, the non-HCC patients may have developed other conditions that required high expenditures, which would have contributed to these observations.

This study has several limitations. First, our database did not include information on cancer staging. Few studies have estimated HCC-associated healthcare expenditures according to stage [4,28]. By analyzing SEER-Medicare linked data, White et al. found that expenditures increased with advancing stage, with additional expenditures of $7,265 for localized HCC, $8,072 for regional HCC, and $9,585 for distant HCC [28]. Stage-specific costing parameters are needed for studies on the cost-effectiveness of treatments (e.g., anticancer agents) in each disease stage. In contrast, estimates based on overall costing parameters, such as those provided in this study, are likely to be adequate for general budget impact analyses or cost-effectiveness analyses aimed at reducing HCC incidence. Second, our subjects were limited to new cases of primary HCC. Because HCC can reoccur after treatment, our estimates do not necessarily represent real-world scenarios. However, if recurrent HCC cases were included in the analysis, the initial phase of the recurrent HCC would overlap with the continuing phase of the initial HCC, resulting in an overestimation of expenditures in the continuing phase. Our study therefore focused only on new cases of HCC to remove this form of bias. Nevertheless, recurrence is a key stage of cancer progression, and analyses that aim to quantify all HCC-associated expenditures should consider the inclusion of additional costs incurred by recurrent cases. Third, the matched cases in our analysis had generally higher disease severity than the unmatched cases across the disease phases. Therefore, our estimates may be biased toward more severe cases. Fourth, our study was limited to HCC patients, and the findings are therefore not generalizable to other diseases. However, our study examined 15 treatment combinations for HCC patients and produced estimates according to disease phase. This approach therefore covers a wide variety of treatments and disease conditions. Nevertheless, future studies should also be conducted on chronic diseases such as diabetes and hypertension.

## Conclusion

The matched comparison and fixed-effects analysis produced similar estimates of HCC-associated healthcare expenditures in the initial and continuing phases, suggesting that either method can be used for these phases depending on data type and availability. However, the estimates diverged substantially in the terminal phase between the methods, and these expenditures should be calculated using matched comparisons.

## References

[1] Ferlay J, Soerjomataram I, Dikshit R, et al Cancer incidence and mortality worldwide: sources, methods and major patterns in GLOBOCAN 2012. *Int J Cancer* 2015;136:E359–86. 10.1002/ijc.29210 25220842

[2] Bruix J, Sherman M. Management of hepatocellular carcinoma: an update. *Hepatology* 2011;53:1020–2. 10.1002/hep.24199 21374666 PMC3084991

[3] El-Serag HB. Hepatocellular carcinoma. *N Engl J Med* 2011;365:1118–27. 10.1056/NEJMra1001683 21992124

[4] Lang K, Danchenko N, Gondek K, Shah S, Thompson D. The burden of illness associated with hepatocellular carcinoma in the United States. *J Hepatol* 2009;50:89–99. 10.1016/j.jhep.2008.07.029 18977551

[5] Tapper EB, Parikh ND. Mortality due to cirrhosis and liver cancer in the United States, 1999–2016: observational study. *BMJ* 2018;362:k2817 10.1136/bmj.k2817 30021785 PMC6050518

[6] Ministry of Health, Labour and Welfare. Vital statistics. Available from: https://www.mhlw.go.jp/english/database/db-hw/vs01.html. Accessed March 20, 2019.

[7] Chahal HS, Marseille EA, Tice JA, et al Cost-effectiveness of Early Treatment of Hepatitis C Virus Genotype 1 by Stage of Liver Fibrosis in a US Treatment-Naive Population. *JAMA Intern Med* 2016;176:65–73. 26595724 10.1001/jamainternmed.2015.6011 PMC5144154

[8] Chhatwal J, Kanwal F, Roberts MS, Dunn MA. Cost-effectiveness and budget impact of hepatitis C virus treatment with sofosbuvir and ledipasvir in the United States. *Ann Intern Med* 2015;162:397–406. 10.7326/M14-1336 25775312 PMC4435698

[9] Chidi AP, Rogal S, Bryce CL, et al Cost-effectiveness of new antiviral regimens for treatment-naïve U.S. veterans with hepatitis C. *Hepatology* 2016;63:428–36. 10.1002/hep.28327 26524695 PMC4718749

[10] Najafzadeh M, Andersson K, Shrank WH, et al Cost-effectiveness of novel regimens for the treatment of hepatitis C virus. *Ann Intern Med* 2015;162:407–19. 10.7326/M14-1152 25775313

[11] Younossi ZM, Park H, Saab S, Ahmed A, Dieterich D, Gordon SC. Cost-effectiveness of all-oral ledipasvir/sofosbuvir regimens in patients with chronic hepatitis C virus genotype 1 infection. *Aliment Pharmacol Ther* 2015;41:544–63. 10.1111/apt.13081 25619871

[12] Sarasin FP, Giostra E, Hadengue A. Cost-effectiveness of screening for detection of small hepatocellular carcinoma in western patients with Child-Pugh class A cirrhosis. *Am J Med* 1996;101:422–34. 10.1016/S0002-9343(96)00197-0 8873514

[13] Arguedas MR, Chen VK, Eloubeidi MA, Fallon MB. Screening for hepatocellular carcinoma in patients with hepatitis C cirrhosis: a cost-utility analysis. *Am J Gastroenterol* 2003;98:679–90. 10.1111/j.1572-0241.2003.07327.x 12650806

[14] Lin OS, Keeffe EB, Sanders GD, Owens DK. Cost-effectiveness of screening for hepatocellular carcinoma in patients with cirrhosis due to chronic hepatitis C. *Aliment Pharmacol Ther* 2004;19:1159–72. 10.1111/j.1365-2036.2004.01963.x 15153169

[15] Patel D, Terrault NA, Yao FY, Bass NM, Ladabaum U. Cost-effectiveness of hepatocellular carcinoma surveillance in patients with hepatitis C virus-related cirrhosis. *Clin Gastroenterol Hepatol* 2005;3:75–84. 10.1016/s1542-3565(04)00443-4 15645408

[16] Tanaka H, Iijima H, Nouso K, et al Cost-effectiveness analysis on the surveillance for hepatocellular carcinoma in liver cirrhosis patients using contrast-enhanced ultrasonography. *Hepatol Res* 2012;42:376–84. 10.1111/j.1872-034X.2011.00936.x 22221694

[17] Cadier B, Bulsei J, Nahon P, et al Early detection and curative treatment of hepatocellular carcinoma: A cost-effectiveness analysis in France and in the United States. *Hepatology* 2017;65:1237–1248. 10.1002/hep.28961 28176349

[18] Yabroff KR, Lamont EB, Mariotto A, et al Cost of care for elderly cancer patients in the United States. *J Natl Cancer Inst* 2008;100:630–41. 10.1093/jnci/djn103 18445825

[19] Thein HH, Isaranuwatchai W, Campitelli MA, et al Health care costs associated with hepatocellular carcinoma: a population-based study. *Hepatology* 2013;58:1375–84. 10.1002/hep.26231 23300063

[20] Thein HH, Qiao Y, Young SK, et al Trends in health care utilization and costs attributable to hepatocellular carcinoma, 2002–2009: a population-based cohort study. *Curr Oncol* 2016;23:e196–220. 10.3747/co.23.2956 27330357 PMC4900840

[21] Vitor S, Marinho RT, Gíria J, Velosa J. An observational study of the direct costs related to hospital admissions, mortality and premature death associated with liver disease in Portugal. *BMC Res Notes* 2016;9:62 10.1186/s13104-016-1879-8 26843372 PMC4739395

[22] Jinjuvadia R, Salami A, Lenhart A, Jinjuvadia K, Liangpunsakul S, Salgia R. Hepatocellular Carcinoma: A Decade of Hospitalizations and Financial Burden in the United States. *Am J Med Sci* 2017;354:362–369. 10.1016/j.amjms.2017.05.016 29078840 PMC5986560

[23] Kaplan DE, Chapko MK, Mehta R, et al Healthcare Costs Related to Treatment of Hepatocellular Carcinoma Among Veterans with Cirrhosis in the United States. *Clin Gastroenterol Hepatol* 2018;16:106–114.e5. 10.1016/j.cgh.2017.07.024 28756056 PMC5735018

[24] Stepanova M, De Avila L, Afendy M, et al Direct and Indirect Economic Burden of Chronic Liver Disease in the United States. *Clin Gastroenterol Hepatol* 2017;15:759–766.e5. 10.1016/j.cgh.2016.07.020 27464590

[25] Lang HC1, Wu JC, Yen SH, Lan CF, Wu SL. The lifetime cost of hepatocellular carcinoma: a claims data analysis from a medical centre in Taiwan. *Appl Health Econ Health Policy* 2008;6:55–65. 10.2165/00148365-200806010-00005 18774870

[26] McAdam-Marx C, McGarry LJ, Hane CA, Biskupiak J, Deniz B, Brixner DI. All-cause and incremental per patient per year cost associated with chronic hepatitis C virus and associated liver complications in the United States: a managed care perspective. *J Manag Care Pharm* 2011;17:531–46. 10.18553/jmcp.2011.17.7.531 21870894 PMC10438304

[27] Menzin J, White LA, Nichols C, Deniz B. The economic burden of advanced liver disease among patients with hepatitis C virus: a large state Medicaid perspective. *BMC Health Serv Res* 2012;12:459 10.1186/1472-6963-12-459 23241078 PMC3529684

[28] White LA, Menzin J, Korn JR, Friedman M, Lang K, Ray S. Medical care costs and survival associated with hepatocellular carcinoma among the elderly. *Clin Gastroenterol Hepatol* 2012;10:547–54. 10.1016/j.cgh.2011.12.031 22210536

[29] Rein DB, Borton J, Liffmann DK, Wittenborn JS. The burden of hepatitis C to the United States Medicare system in 2009: Descriptive and economic characteristics. *Hepatology* 2016;63:1135–44. 10.1002/hep.28430 26707033

[30] Golabi P, Jeffers T, Younoszai Z, et al Independent Predictors of Mortality and Resource Utilization in Viral Hepatitis Related Hepatocellular Carcinoma. *Ann Hepatol* 2017;16:555–564. 10.5604/01.3001.0010.0290 28611258

